# Experimental Validation of a Battery-Free RFID-Powered Implantable Neural Sensor and Stimulator

**DOI:** 10.3390/s26030954

**Published:** 2026-02-02

**Authors:** Luís Eduardo Pedigoni Bulisani, Marco Antonio Herculano, Carolina Chen Pauris, Luma Rissatti Borges do Prado, Lucas Jun Sakai, Francisco Martins Portelinha Júnior, Evaldo Marchi

**Affiliations:** 1 Department of Research and Postgraduate Studies, Jundiaí Medical School, Rua Francisco Telles, 250, Vila Arens, Jundiaí 13202-550, SP, Brazil; 2 Health Laboratory, National Institute of Telecommunications (Inatel), Av. João de Camargo, 510, Centro, Santa Rita do Sapucaí 37536-001, MG, Brazil

**Keywords:** bioelectronic medicine, neuroprosthetics, battery-free implantable devices, wireless power transfer, neural interfaces, peripheral nerve stimulation, RFID-powered systems

## Abstract

Introduction: Neurological injuries significantly impair quality of life by disrupting neural transmission. Traditional implantable stimulators often rely on internal batteries, which limit device longevity and necessitate repeated surgical interventions. Objective: This study presents the experimental validation of a battery-free, RFID-powered neural platform for peripheral nerve signal acquisition and stimulation, targeting TRL-6 validation. Methods: The prototype incorporates an adjustable analog front-end with gains up to 93 dB and a biphasic current-controlled stimulator. Validation was performed through benchtop testing, biological tissue assessments using porcine tissue, and functional in vivo trials in adult Wistar rats (n = 3) over a three-month period. Results: Benchtop evaluation confirmed gain accuracy with errors below 2.2 dB and precise stimulation timing. The system maintained a stable 3.3 V wireless power link through 20 mm of biological tissue using RFID. In vivo experiments indicated a 100% functional success rate (51/51 trials) in eliciting gross motor responses via wireless stimulation. Thermal safety was confirmed, with a maximum operating temperature of 28 °C, remaining well below physiological limits. Conclusions: The results demonstrate the functional feasibility of a battery-free, RFID-powered neural interface for wireless signal acquisition and stimulation, supporting system-level validation of this architecture.

## 1. Introduction

Peripheral nerve injuries commonly result from trauma and lead to progressive loss of motor and sensory function, often accompanied by severe muscle atrophy and chronic disability [1]. When these injuries affect the upper limbs, patients may experience a substantial reduction in autonomy, compromising their ability to perform basic daily activities and significantly affecting quality of life [2]. Beyond the initial injury, the lack of sustained neural communication contributes to long-term functional decline, highlighting the need for technologies capable of establishing stable and prolonged interfaces with peripheral nerves.

To address this clinical need, implantable neural interfaces have been increasingly explored as platforms for recording and stimulating bioelectrical signals in the peripheral nervous system, enabling direct interaction between electronic systems and neural tissue. Such devices have applications in neurophysiological research, bioelectronic medicine, and the development of advanced neuroprosthetic and monitoring technologies [3].

From an engineering perspective, the design of implantable neural devices poses significant technical challenges, including long-term power supply, miniaturization, biocompatibility, and reliable wireless communication. Conventional implantable systems typically rely on internal batteries, which limit device lifetime, increase volume, and raise safety concerns related to replacement procedures and long-term implantation. These constraints have motivated growing interest in battery-free implantable architectures powered through wireless energy transfer [4].

Radio-frequency identification (RFID)–based systems have emerged as a promising approach for wireless powering and data communication in implantable and wearable biomedical devices. RFID technology enables energy harvesting and bidirectional communication using compact coils and external readers, making it particularly attractive for miniaturized implantable sensors and stimulators [5]. Previous studies have demonstrated RFID-powered devices for physiological monitoring [6]; however, the integration of wireless power harvesting, neural signal acquisition, and electrical stimulation within a single implantable platform remains technically challenging.

In parallel, peripheral nerve interfaces often require simultaneous recording and stimulation capabilities to enable closed-loop or bidirectional interaction with neural pathways [7]. Achieving stable signal acquisition while delivering electrical stimulation in a compact, battery-free system demands careful system-level design, including front-end circuitry, power management, and signal integrity optimization.

Recent work on implantable neural interfaces has explored both rechargeable and battery-free architectures for peripheral nerve recording and stimulation, encompassing academic and translational developments with different design trade-offs in terms of power delivery, miniaturization, and functional integration [3,4,5,6,7]. Although certain approaches prioritize multi-channel configurations for specific neuroprosthetic applications, battery-free systems often focus on validating core functionalities under strict power and size constraints. Accordingly, recent battery-free implantable approaches have increasingly focused on demonstrating the integration of wireless power harvesting, neural signal acquisition, and electrical stimulation within a single compact platform [4,5,6,7]. Beyond standalone sensing and stimulation, bidirectional peripheral nerve interfaces have the potential to enable advanced applications such as closed-loop neuroprosthetic control and neural bridging strategies, including the actuation of robotic or assistive devices.

In this context, this study presents the design and experimental validation of a battery-free, RFID-powered implantable neural sensor and stimulator. The proposed system integrates wireless power harvesting, neural signal acquisition, and electrical stimulation within a miniaturized implantable platform, supported by an external unit for control and data processing. By eliminating the need for internal batteries, this architecture addresses the critical challenges of device longevity and surgical risks associated with battery replacement, offering a sustainable path for long-term neuroprosthetic and bioelectronic interfaces.

The objective of this study is to experimentally validate a battery-free, RFID-powered implantable neural device by assessing three core system-level functions: wireless power harvesting, bioelectrical signal acquisition, and controlled electrical stimulation.

## 2. Materials and Methods

### 2.1. System Architecture and Engineering Validation Plan

A battery-free implantable prototype designed for peripheral nerve signal acquisition and electrical stimulation was developed over a three-year period through iterative laboratory testing at the National Institute of Telecommunications (INATEL, MG, Brazil). The present study describes the experimental procedures used to evaluate the prototype at the TRL-6 stage, with emphasis on system-level engineering performance under relevant experimental conditions [8].

**Figure 1:**
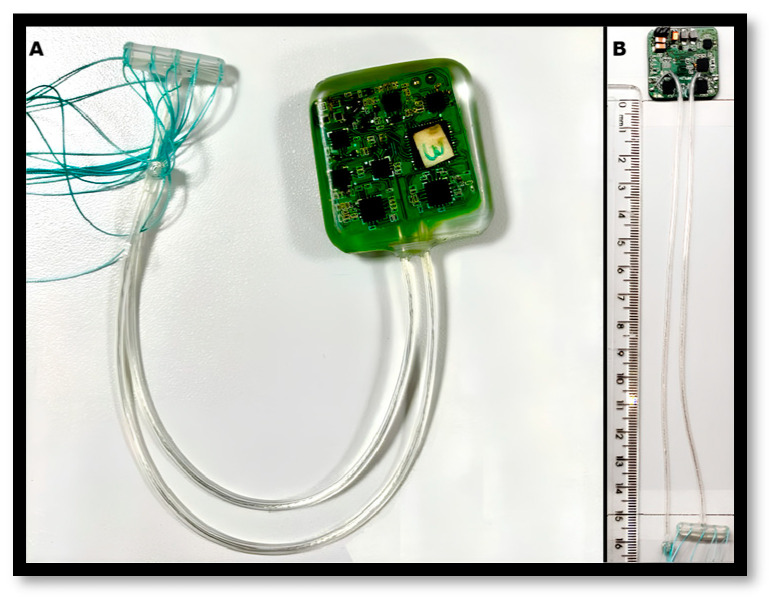
(**A**) Encapsulated implant connected to two commercially available extraneural cuff electrodes with distinct functional roles, where the larger electrode is dedicated to neural signal acquisition and the smaller electrode delivers electrical stimulation. The device is encapsulated in a transparent biocompatible epoxy resin, and the attached sutures facilitate secure placement of the electrodes around the nerve. (**B**) Photograph of the implantable unit and connecting leads positioned alongside a millimeter-scale ruler to provide a dimensional reference.

The implantable device (ID) integrates neural signal acquisition and electrical stimulation within a compact form factor, with approximate dimensions of 30 mm × 30 mm × 15 mm, comparable to implantable pulse generators (IPGs). The device is encapsulated in a transparent biocompatible epoxy resin (Biokyra EB-130M-1, Epoxyset Inc., Woonsocket, RI, USA), compliant with ISO 10993 standards and certified by the manufacturer for long-term implantation exceeding 30 days (Figure 1) [9].

The ID incorporates two extraneural cuff electrodes: one dedicated to neural signal acquisition and the other to electrical stimulation. Commercial Standard Nerve Cuff electrodes (MicroProbes for Life Science, Inc., Gaithersburg, MD, USA) were employed to ensure reproducibility and standardization of the nerve interface. The recording electrode consisted of a 2 mm inner diameter cuff with four 250 µm platinum contacts, while the stimulation electrode consisted of a 1.5 mm inner diameter cuff with three 250 µm platinum contacts. Both electrodes featured silicone insulation, customized spacing with sutures, and 15 cm lead lengths. According to manufacturer specifications, the typical impedance of each 250 µm platinum/iridium contact is approximately 1–3 kΩ at 1 kHz. In this context, the term ‘electrodes’ refers to distinct cuff devices assigned to recording and stimulation, while ‘contacts’ refer to the individual platinum interfaces within each cuff that define the electrical interaction with the nerve.

The current prototype implements one dedicated neural acquisition channel and one dedicated electrical stimulation channel. For signal acquisition, the four platinum contacts of the recording cuff are connected to the analog front-end, which performs differential sensing and signal conditioning prior to digitization, resulting in a single acquisition channel. Neural stimulation is delivered through the multi-contact cuff electrode using defined positive, negative, and reference contacts to generate symmetric biphasic stimulation pulses, rather than applying the same waveform simultaneously to all platinum contacts. These two functional paths are realized through separate analog front-end and stimulation circuits, sharing the same wireless power and bidirectional communication interface.

Commercial extraneural cuff electrodes were intentionally selected to minimize variability related to electrode design and implantation geometry, allowing the in vivo evaluation to focus on validation of the wireless sensing and stimulation architecture rather than on electrode performance or physiological characterization [10].

**Figure 2:**
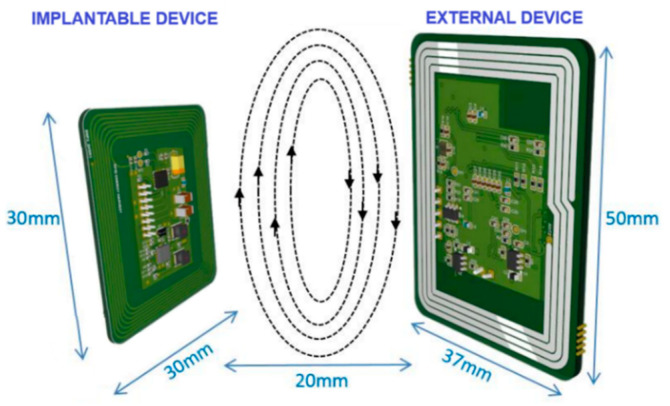
Illustration of the dimensions of the implantable and external devices, highlighting their respective antennas and bidirectional RFID connection. The central arrows indicate the bidirectional wireless power and data link between devices. The devices operate with a maximum separation of 20 mm. The PCB layout was designed using Altium Designer (version 24, Altium LLC, San Diego, CA, USA).

The implantable system operates at a carrier frequency of 13.56 MHz, within the internationally allocated industrial, scientific, and medical (ISM) band [11,12]. Wireless power transfer and bidirectional data communication between the implantable device and an external unit are achieved using passive radio-frequency identification (RFID) technology (Figure 2). Benchtop and tissue-based tests were conducted using biological tissue analogs, including porcine and chicken tissue, to evaluate wireless power transfer and data communication (Supplementary Figure S7).

The wireless front-end is responsible for inductive energy harvesting, RF field modulation and demodulation, and protocol-level frame handling according to the ISO/IEC 14443 standard [13]. Harvested RF energy is rectified and regulated to supply the implant’s digital and analog circuits in real time, without local energy storage. Bidirectional communication between the wireless front-end and the internal control unit is performed via a serial digital interface, enabling command reception and neural data transmission through the same HF link.

The wireless front-end employed in the present system relies on a proprietary commercial component that integrates energy harvesting and communication functions. Due to intellectual property and patent-related constraints, the exact commercial identity of this component cannot be disclosed. Nevertheless, its functional role, operating principles, performance characteristics, and system-level integration are described in sufficient detail to support methodological reproducibility within the scope of this study. Accordingly, the reported results are intended to be reproducible using functionally equivalent HF RFID/NFC front-end components, rather than requiring exact duplication of a specific commercial device.

The implant consists of two printed circuit boards (PCBs): one dedicated to the RFID antenna and transponder circuitry, and a second dedicated to signal acquisition and electrical stimulation. Antenna geometry and layout were optimized to support device miniaturization while maintaining stable wireless coupling at the operating frequency.

In passive RFID operation, the implantable device harvests energy from the electromagnetic field generated by the external reader, without the use of an internal battery [14]. The external unit supplies radio-frequency power, controls device operation, and processes data transmitted from the implant. The external unit is powered by a rechargeable battery and provides visual indicators for battery status and wireless link integrity. Bluetooth communication is used to transmit data from the external unit to a computer-based user interface, where signal visualization and parameter control are performed using custom firmware.

The implantable device operates exclusively in a strictly real-time powered, battery-free mode using passive RFID energy harvesting. Device operation is fully dependent on the presence of the external RF field and ceases immediately when the wireless link is interrupted. The system was designed to maintain a regulated 3.3 V supply at a 20 mm separation through biological tissue. Under these conditions, current consumption was characterized across the full operational range, from approximately 0.5 mA in idle mode to 3.6 mA during concurrent active acquisition and stimulation.

The signal acquisition circuitry within the implantable device is designed to amplify and filter electroneurographic (ENG) signals prior to digitization. The analog front-end provides a programmable total gain ranging from approximately 63 dB (1400×) to 93 dB (45,000×), adjustable in 2 dB increments. The amplification chain consists of a pre-amplifier stage (103×), followed by two adjustable gain stages with gains ranging from 1.35× to 21× and from 10× to 21×, respectively. Signal conditioning is achieved using cascaded second-order Butterworth filters, including a high-pass filter with a 500 Hz cutoff frequency and a low-pass filter with a 5000 Hz cutoff frequency. Capacitive coupling is employed at the input stage to eliminate DC offsets. Residual baseline variations arising from environmental noise or transient artifacts may be present but are not considered physiologically meaningful within the scope of signal peak detection. Analog-to-digital conversion is performed with adjustable resolutions ranging from 6 to 12 bits and selectable sampling rates of 3.125, 4, 5, 6.25, 8, 10, or 12.5 ksps. Power supply stabilization is achieved using passive filtering components, without local energy storage elements [15,16].

The implantable device also incorporates an electrical stimulation circuit capable of generating biphasic stimulation waveforms. Stimulation signals are produced by the microcontroller’s digital-to-analog converter (DAC) and converted to controlled current output through a constant-current source. The circuit is designed to deliver controlled current output for load impedances up to 5 kΩ, accounting for variations associated with electrode–tissue interfaces. A current monitoring circuit provides feedback to the microcontroller to ensure adherence to programmed current limits during stimulation [16,17].

During benchtop testing, wireless power transfer and communication were evaluated using biological tissue analogs and device encapsulation materials. A power conversion stage provides regulated +5 V and −5 V supply rails for the implantable device circuits. Power management in the implantable device occurs at the wireless interface stage, where RF energy harvested from the external unit is rectified and regulated by a dedicated power conversion stage to supply the internal analog and digital circuits. This configuration is compliant with Near Field Communication (NFC) standards, characterizing a short-range high-frequency RFID system rather than a UHF-based architecture.

Programmable stimulation parameters include current amplitude (100–1200 µA), pulse width (100–250 µs), frequency (10–30 Hz), and stimulation duration (500 ms to 25 s). The maximum injected charge per phase (300 nC, corresponding to 1200 µA and 250 µs) was defined based on reported safety limits for peripheral nerve stimulation [18]. Stimulation waveforms are generated by the microcontroller and delivered through a constant-current source circuit [19].

The implantable prototype implemented in this study supports neural signal acquisition and electrical stimulation through a bidirectional wireless interface, allowing neural signals to be transmitted to an external unit for processing and, when required, stimulation commands to be sent back to the implant, while prioritizing low power consumption and system simplicity.

System validation was conducted in three sequential stages: benchtop electrical characterization of signal acquisition and stimulation circuits, validation of wireless power transfer and data communication through biological tissue analogs, and in vivo functional validation of signal acquisition and stimulation. Due to the high production cost of the prototypes, the evaluation was limited to three devices.

### 2.2. Experimental Validation Protocol

#### 2.2.1. Benchtop Electrical Characterization

Benchtop electrical characterization was conducted to verify the functionality of the implantable device under controlled laboratory conditions prior to tissue-based and in vivo experiments. This validation stage was designed to assess the operation of the signal acquisition and electrical stimulation subsystems independently of biological variability.

The signal acquisition pathway was evaluated by applying calibrated electrical test signals directly to the recording input terminals. These signals were generated to emulate peripheral electroneurographic (ENG) amplitudes and frequency content. Output data were transmitted wirelessly to the external unit and visualized through the computer-based interface to verify amplification, filtering, digitization, and data transmission across the programmable gain and sampling configurations.

Electrical stimulation performance was characterized using resistive loads to emulate electrode–tissue impedance. A precision resistor (1 kΩ) was connected to the stimulation output, and biphasic stimulation waveforms were generated using predefined combinations of current amplitude, pulse width, frequency, and stimulation duration. Voltage waveforms across the load were monitored using a digital oscilloscope to verify pulse shape and timing consistency with programmed parameters.

All benchtop experiments were performed using the same firmware and configuration settings later employed during tissue-based and in vivo validation stages, ensuring continuity across the experimental workflow. Additional electrical characterization data are provided in the Supplementary Materials.

**Figure 3:**
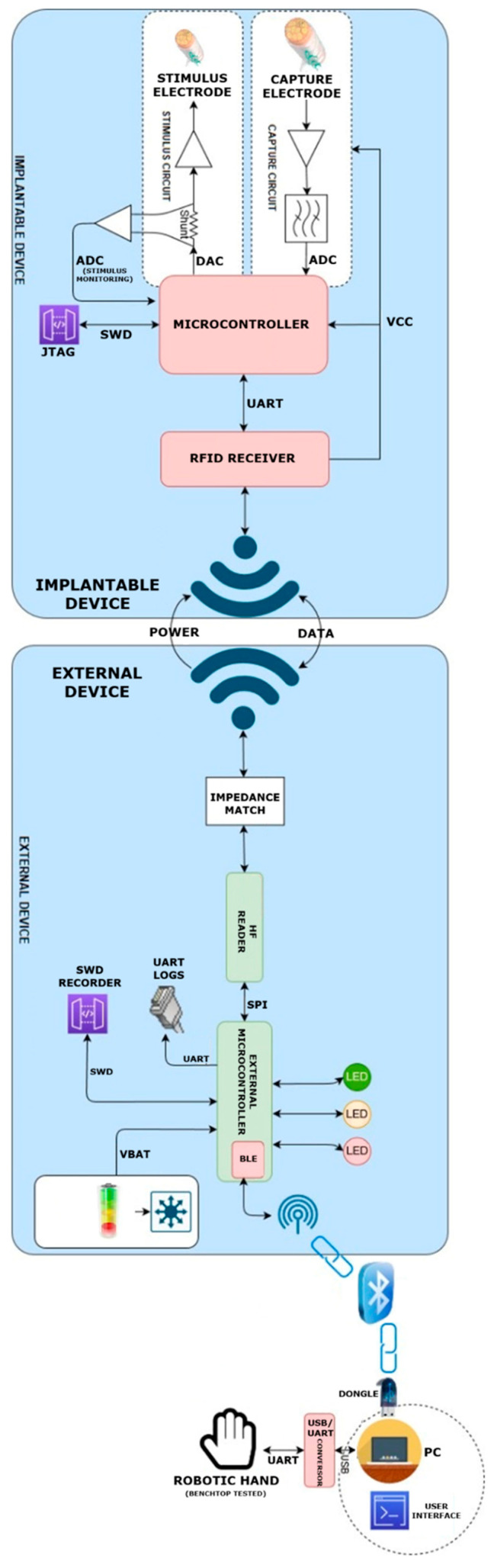
System-level schematic of the implantable and external devices. The implant records and stimulates peripheral nerves and communicates wirelessly with the external unit via RFID for both data and power transfer. The external unit interfaces with a computer-based system and optional external actuators (e.g., robotic hand). This architecture was used consistently during benchtop, tissue-based, and in vivo experimental protocols. The schematic was created using draw.io (version 26.1.3, JGraph Ltd., Northampton, UK).

Bioelectrical signals are captured by the implantable device through the recording interface, processed by the internal acquisition circuitry, and transmitted wirelessly to an external unit via an RFID-based communication link. The external unit interfaces with a computer-based system used for signal visualization, parameter configuration, and firmware control. This system architecture was consistently employed across benchtop, tissue-based, and in vivo experimental stages (Figure 3).

#### 2.2.2. Wireless Operation Through Biological Tissue

Wireless operation through biological tissue was assessed under conditions that approximate in vivo signal propagation and electromagnetic attenuation. This validation stage was conducted following benchtop electrical characterization and prior to animal implantation, allowing isolation of tissue-related effects on wireless power transfer and data communication.

Tissue-based tests were performed using ex vivo biological tissue analogs, including porcine and chicken tissue, selected to represent heterogeneous soft-tissue compositions commonly encountered in superficial implantation scenarios. The implantable device was positioned beneath the tissue samples, while the external unit was aligned parallel to the implant to establish RFID-based power transfer and bidirectional communication.

During testing, the implantable device operated exclusively in battery-free mode, powered through the RFID link. Wireless communication stability was evaluated while performing signal acquisition and stimulation triggering under the same firmware and configuration settings used during benchtop characterization. Device operation was verified by monitoring successful data transmission to the external unit and subsequent visualization via the computer-based interface. Representative tissue-based wireless operation tests are presented in the Supplementary Materials.

In addition to wireless power and data validation through biological tissue, a system-level integration test was performed using the same tissue-based setup. In this configuration, predefined externally generated electrical test signals were applied to the implant system under tissue loading. These signals were processed by the embedded firmware, which generated discrete digital trigger commands. The commands were transmitted wirelessly to the external unit and forwarded via a serial interface (UART) to an external robotic hand controller, producing opening and closing movements. This experiment was designed to verify end-to-end system integration under tissue attenuation conditions, including wireless transmission, firmware operation, and external command execution.

All tissue-based experiments were conducted using the same hardware configuration employed in subsequent in vivo studies, ensuring consistency across validation stages and enabling direct comparison between laboratory and biological testing conditions.

#### 2.2.3. In Vivo Functional Validation

Three adult Wistar rats (*Rattus norvegicus*), with an average body weight of 735 g, were used in this study. The animals were obtained from the central animal facility of the University of Campinas (São Paulo, Brazil), and all experimental procedures were conducted at the Jundiaí Medical School. All implantation procedures were performed in a dedicated surgical room under sterile conditions. Anesthesia was induced using an intramuscular injection of ketamine (60 mg/kg; Quetamina^®^, Vetnil^®^, Louveira, SP, Brazil) and xylazine (1 mg/kg; Sedanew^®^ 2%, Vetnil^®^, Louveira, SP, Brazil), administered at a volume of 0.1 mL per 100 g of body weight into the gluteus maximus muscle. After confirmation of adequate anesthesia, the animals were positioned in left lateral recumbency for trichotomy and antisepsis of the right hind limb using 2% chlorhexidine digluconate, followed by ventral recumbency for trichotomy and antisepsis of the dorsal region.

A longitudinal skin incision was performed in the posterior thigh to expose the biceps femoris muscle, which was gently retracted to allow identification of the sciatic nerve. A second longitudinal incision was made in the paravertebral region to create a subcutaneous pocket for implantation. A subcutaneous tunnel connecting the dorsal thoracic incision to the posterior thigh incision was formed using blunt dissection, allowing passage of the electrode leads from the implantation site to the target nerve region.

**Figure 4:**
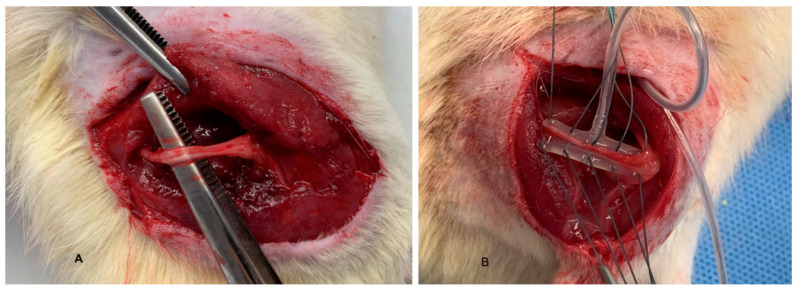
(**A**) Isolation of the sciatic nerve. (**B**) Placement of the recording electrode on the sciatic nerve.

**Figure 5:**
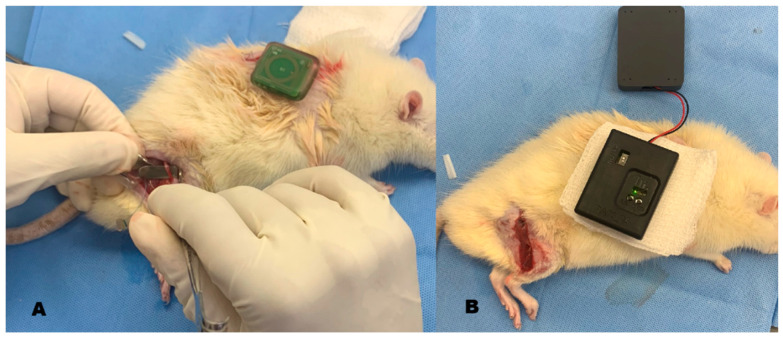
(**A**) Isolation of the sciatic nerve and electrode placement. The image shows incisions in the posterior thigh and dorsal thoracic regions. The electrodes were tunneled subcutaneously to the implantation site (dorsal region), where the battery-free internal device was implanted. (**B**) Communication test between the implanted device and the external unit (Black boxes). The lower black box contains the RFID and Bluetooth modules and is positioned parallel to the implant, while the upper box houses the battery. The green LED is lit, used as an operational indicator of wireless link availability. The same implantable coil antenna illustrated in Figure 2 was used in this experiment.

Following dissection of the sciatic and anterior tibial nerves, the recording cuff electrode was positioned around the sciatic nerve, and the stimulation cuff electrode was placed around the anterior tibial nerve, maintaining an approximate spacing of 1 cm between electrodes. The cuff electrodes, equipped with integrated sutures, were placed circumferentially around the nerves without applying compressive force. The electrodes were implanted at an estimated depth of 5–7 mm from the skin surface (Figure 4). The battery-free implantable device was positioned on the dorsal region and placed subcutaneously at a depth of approximately 3–4 mm (Figure 5A).

Following implantation, the external unit was positioned externally and aligned parallel to the implanted device to establish RFID-based power transfer and data communication. An integrated visual indicator (LED) on the external unit was used as an operational indicator of wireless communication readiness prior to subsequent testing (Figure 5B).

After completion of the implantation procedure, soft tissues were repositioned according to anatomical stratification and closed using 4-0 nylon sutures (Technofio^®^, Ace Ind. e Com. Ltda, Goiânia, GO, Brazil). Postoperative care included a subcutaneous dose of enrofloxacin (0.05 mg/100 g; Chemitril^®^ Injectable 10%, Chemitec, São Paulo, SP, Brazil) and tramadol hydrochloride (0.05 mg/100 g) administered immediately after surgery. During the postoperative period, enrofloxacin was administered daily for seven days, and tramadol hydrochloride was administered for three days. Dipyrone was diluted in the animals’ drinking water and provided for seven postoperative days.

All animal care and experimental procedures were conducted in accordance with the ARRIVE guidelines and were approved by the Ethics Committee for Animal Use (CEUA) of the Jundiaí Medical School (approval number 22538137). Animals were housed under a 12 h light/dark cycle with ad libitum access to food and water in the animal facility of the Jundiaí Medical School. All procedures complied with relevant institutional, national, and international regulations governing the ethical use of animals in research.

Following recovery, animals were monitored for a period of three months. Experimental data were recorded during scheduled testing sessions using software specifically developed to interface with the implantable device. During these sessions, the external unit was temporarily affixed to the animal’s back using adhesive tape and positioned parallel to the implanted device to enable wireless power transfer and communication via the RFID interface. The external unit was removed outside scheduled testing sessions. 

No euthanasia procedure was performed at the end of the study period. All experimental protocols complied with the AVMA Guidelines for the Euthanasia of Animals (2020) and the Brazilian CONCEA and CEUA regulations (protocol 22538137). Following completion of the experimental protocol, the animals were relocated to the animal facility of the Jundiaí School of Medicine and were not subjected to additional injuries, experimental procedures, or euthanasia.

During the monitoring period, experimental sessions were conducted to assess bioelectrical signal acquisition and electrical stimulation using the implanted device. Sessions were performed on scheduled days, with animals evaluated individually. During each session, the external unit was temporarily positioned parallel to the implanted device to enable wireless power transfer and data communication via the RFID interface. The external unit was connected only during active testing sessions and removed immediately afterward. A comprehensive experimental approach was adopted to evaluate the peripheral signal capture interface under controlled conditions, focusing on its response to standardized tactile stimulation protocols.

Bioelectrical activity was recorded using the implanted acquisition interface while controlled tactile stimulation was applied to the plantar surface of the hind paw using a mechanical stimulus comparable to a Von Frey filament, a well-established method for assessing peripheral sensitivity and stimulus–response association [20]. Electrophysiological recordings were obtained concurrently with stimulation and transmitted wirelessly to the external unit and computer-based system for visualization and storage.

Electrical stimulation was delivered through the implanted stimulation electrode using programmable, current-controlled biphasic pulses generated by the implantable device microcontroller upon receiving control commands from the external unit [21]. Biphasic waveforms were selected to minimize electrochemical reactions at the electrode–tissue interface and reduce the risk of tissue damage, in accordance with commonly adopted practices in peripheral nerve stimulation [22].

Specific absorption rate (SAR) testing was not performed, as it was outside the scope of the present study, which focused on validating the functional operation of the prototype. The RFID carrier frequency employed is considered safe according to established medical and engineering literature [23]. Detailed acquisition parameters, stimulation configurations, safety considerations, and representative recordings are provided in the Supplementary Materials.

## 3. Results

Prior to integrated system testing, core hardware performance was validated through benchtop gain characterization. As summarized in Table 1, theoretical and measured gain values were assessed across two representative operational settings (63 dB and 93 dB) using three device interfaces (D1–D3). The measured linear and decibel gains closely matched the theoretical values, with error margins as low as 0.67 dB for the 63 dB configuration. These results indicated stable and reliable performance of the analog acquisition chain prior to biological testing.

The system’s ability to handle low-amplitude signals was further characterized through integrated benchtop tests using a simulated 1 kHz nerve signal. Signal integrity across the analog acquisition chain was verified under a 60 dB attenuation condition, confirming that bioelectrical events exceeding the 40 µV threshold could be reliably detected above the system’s noise floor. Communication robustness was additionally evaluated using biological tissue (porcine muscle and adipose layers) as a transmission medium. Under these conditions, the RFID link maintained stable power delivery (3.3 V) and data transfer at distances of up to 20 mm through tissue, with the external unit providing real-time visual feedback of connection status. This value refers to a regulated DC supply used for digital logic and control, distinct from the ±5 V DC rails employed for analog signal acquisition and stimulation circuitry. In both benchtop and tissue-based trials, the supply remained stable within a ±0.05 V range during all recorded stimulation cycles, indicating stable power delivery under maximum load conditions. The 40 µV threshold was empirically determined during benchtop characterization and early in vivo pilot testing as a conservative level to reliably distinguish evoked bioelectrical activity from the system’s baseline electronic noise floor, ensuring stable triggering without false positives. Because the implant operates in a strictly battery-free, real-time powered mode, stable supply voltage during stimulation implicitly confirms continuous wireless power transfer and communication link integrity throughout the experiment.

The stimulation circuitry was similarly characterized to ensure accurate and repeatable delivery of electrical pulses. As shown in Table 2, system output was evaluated across a range of programmed amplitudes (100–1200 µA), frequencies (10–30 Hz), and pulse widths (100–250 µs) using a 1 kΩ resistive load. Oscilloscope measurements confirmed high temporal and amplitude fidelity; for example, a programmed pulse width of 500 µs was measured at 502 µs, and a 10 s stimulation window was executed in 10.04 s. These results supported that the battery-free stimulator maintained close adherence to programmed parameters, supporting its use in subsequent in vivo functional tests.

Detailed theoretical and measured gain and stimulation results for the amplifier stages are provided in Table 1 and Table 2, confirming system accuracy at the hardware level prior to integrated system and biological testing. Detailed benchtop electrical characterization data supporting these results are provided in Supplementary Figures S1–S6.

Following initial hardware characterization, additional benchtop tests were conducted using biological tissues to assess device performance under conditions closer to real–world applications. Porcine muscle and adipose tissue layers were used to evaluate the influence of tissue on signal transmission and to validate the communication distance between the implantable device (DI) and the external unit (DAPS). As summarized in Supplementary Tables S1–S4 and illustrated by the experimental setup shown in Supplementary Figure S7, the system maintained stable power delivery (3.3 V) under active acquisition and stimulation conditions through tissue thicknesses of up to 22 mm. Qualitative indicators of bidirectional communication under the same configurations are reported in Supplementary Table S5. Accordingly, the main manuscript reports only summary performance metrics, whereas the Supplementary Materials provides full tabulated measurements, oscilloscope traces, and configuration–specific results.

Under tissue–based test conditions, externally generated electrical test signals transmitted through the implantable system produced discrete digital trigger commands that reliably actuated the external robotic hand. Reproducible opening and closing movements were observed, synchronized with transmitted trigger events, confirming correct end–to–end operation of wireless transmission, firmware processing, and external command execution under tissue attenuation. Representative examples of the experimental setup and signal transmission are shown in Supplementary Figures S8 and S9.

Following surgical implantation and post–operative recovery, in vivo experiments indicated successful peripheral electrical signal acquisition and stimulation using the battery–free implant system. Over a three–month monitoring period, the Von Frey sensitivity test (Supplementary Figure S10) consistently elicited paw movement in response to tactile stimulation. A visually apparent temporal association was observed between tactile stimulation, the recorded bioelectrical signal (amplitude > 40 µV), and the corresponding paw extension. 

Concurrent recordings revealed a clear temporal association between detected bioelectrical signals and behavioral responses. Analysis of these signals confirmed the operational performance of the signal capture electrode, demonstrating its ability to consistently detect physiological responses. These results also confirmed successful data transfer between the implanted device and the external unit without the need for an on–board battery, with energy supplied exclusively through RFID–based harvesting.

**Figure 6:**
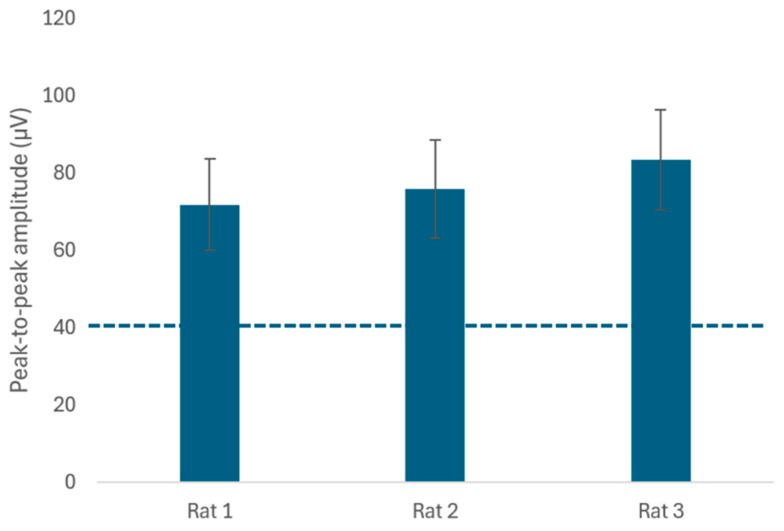
Mean peak–to–peak amplitudes of biosignals recorded from the implanted sensor during behavioral responses to tactile stimulation, summarized per animal as mean ± standard deviation over the 90–day experimental period. The dashed horizontal line denotes the predefined activation threshold (40 µV). Raw session–level data are provided in Supplementary Table S6.

Bioelectrical signals recorded during unscheduled reading tests consistently exceeded the 40 µV threshold during Von Frey stimulation. The quantitative data corresponding to Figure 6 are summarized in Supplementary Table S6. Figure 6 summarizes the mean peak–to–peak amplitudes of bioelectrical signals recorded during tactile stimulation, aggregated per animal across repeated sessions. Session–level measurements were summarized using descriptive statistics to characterize within–subject variability while preserving animals as independent experimental units.

Given the small sample size (n = 3 animals), analyses were limited to descriptive statistics. For each animal, repeated sessions were summarized by mean, median, standard deviation, and coefficient of variation (CV). Animals were treated as experimental units; repeated measures were used to characterize within–subject variability over time. Where informative, simple effect–size or signal–to–noise summaries are reported without hypothesis testing.

Across sessions, fluctuations in the amplitude of the recorded bioelectrical signals were observed, with both increases and decreases occurring without a discernible pattern. The mean and standard deviation for each rat are as follows: Rat 1: Mean = 71.76 µV, Standard Deviation = 11.88 µV; Rat 2: Mean = 75.82 µV, Standard Deviation = 12.75 µV; Rat 3: Mean = 83.47 µV, Standard Deviation = 12.96 µV. The observed amplitude ranges were approximately 66–78 µV for Rat 1, 69–82 µV for Rat 2, and 77–90 µV for Rat 3, indicating stable signal dispersion across sessions. The minimum observed values were 55 µV (for Rats 1 and 2) and 63 µV (for Rat 3).

The coefficient of variation (CV) was calculated for each rat to assess signal stability. Results were: Rat 1 = 16.55 %, Rat 2 = 16.81 %, and Rat 3 = 15.52 %. These values indicate low variability and consistent mean amplitudes above the operational 40 µV threshold, supporting stable implant performance across animals. The observed responses were consistent across repeated sessions, indicating stable device operation.

All reported mean values and dispersion metrics were derived from repeated measurements under identical experimental conditions, providing an assessment of measurement variability and system robustness.

Regarding the stimulation tests, muscle movement was observed in response to controlled stimulation, indicating that the implant was able to evoke a physical response. This stimulation was triggered by a computer command with specific stimulation parameters. Adjusting the stimulation parameters allowed precise control over the strength of the muscle contractions, with higher parameters producing more pronounced leg movements and lower parameters resulting in weaker movements.

Peripheral stimulation tests resulted in 100 % successful responses (51/51 trials), defined as trials in which the implant was powered wirelessly and elicited a clear, reproducible gross motor response (e.g., paw or leg movement) following stimulation. The responses were consistent across animals and sessions, confirming reliable wireless activation and functional delivery of the stimulation waveform.

Qualitative motor effects were consistently observed during tibial nerve stimulation, specifically characterized by synchronized foot dorsiflexion and visible muscle contraction of the hindlimb. While this study did not focus on gait analysis or complex reflex arc quantification, these overt motor responses confirm the stimulator’s efficacy in delivering supra–threshold charge to the target nerve. During stimulation trials, the acquisition system was temporarily blanked to prevent saturation from stimulation artifacts, ensuring signal integrity post–activation.

Importantly, no signs of tissue reaction were observed upon direct visual inspection of the implantation site, and no device malfunction occurred during periods of stable RFID communication (as indicated by the green LED on the external unit). In contrast, when communication was disrupted due to animal movement or adhesive tape detachment, the LED initially turned red, signaling a connection loss, and the device appropriately ceased its functions.

**Figure 7:**
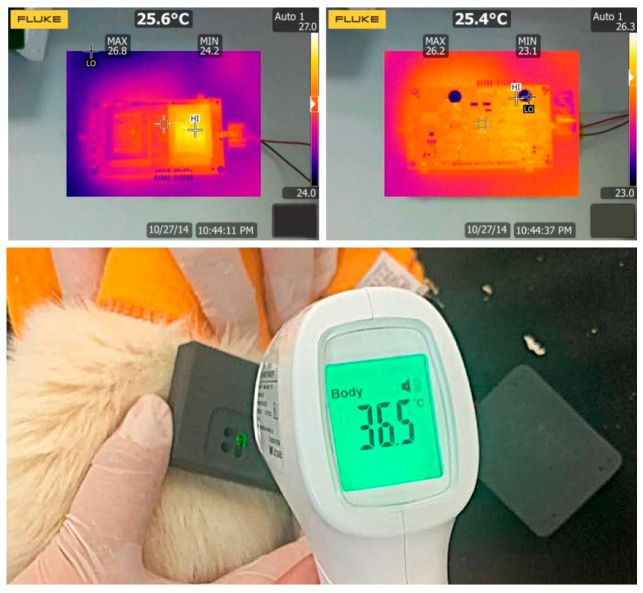
Thermal assessment of the implantable device under different experimental conditions. (**Top**) Infrared thermographic images showing temperature distribution during bench–top testing. (**Bottom**) Temperature measurement following acute in vivo implantation in a rodent model, indicating thermal behavior under physiological conditions.

Thermal safety was evaluated under continuous benchtop operation and during in vivo implantation. Infrared thermographic imaging demonstrated a uniform temperature distribution during active operation (Figure 7). In vivo measurements confirmed that the implanted device remained within normal physiological temperature ranges, maintaining a stable reading of approximately 36.5 °C. These results indicated that the RFID–powered system operated within established biomedical safety margins without inducing thermal stress in surrounding tissues.

## 4. Discussion

This study is positioned as an engineering validation of a battery–free, RFID–powered implantable neural device capable of wireless signal acquisition and stimulation. The primary objective was to demonstrate system–level feasibility, robustness, and functional reliability across benchtop, tissue–based, and in vivo experimental conditions, rather than performing detailed physiological or clinical characterization or focusing on isolated signal events. Within this scope, the results support validation at the TRL–6 stage, emphasizing technical performance, safety, and functional operation in a relevant biological environment. The use of three experimental units was defined by the proof–of–concept nature of this TRL–6 validation, prioritizing reproducibility and consistency of the integrated hardware–tissue interface rather than population–based statistical inference.

Recent wireless implantable systems adopt different architectural tradeoffs between power delivery, data throughput, and functional complexity as summarized in Table 3. Backscatter–based battery–free systems prioritize ultra–low–power operation by focusing on sensing and data communication [5,6]. Platforms such as MobiScatter exemplify this design space, but are typically limited to sensing–only functionality and do not support active neural stimulation. Other RF–powered platforms emphasize improved link robustness and tolerance to coupling variability to enable reliable wireless sensing under practical deployment conditions [24]. Systems such as RFlexor exemplify this design philosophy. In contrast, wireless systems that integrate both recording and stimulation often rely on active power management strategies or local energy buffering, which increases system complexity [17,21]. The present work adopts a different design strategy, prioritizing strictly real–time, battery–free operation using passive RFID power transfer, with simultaneous sensing and stimulation validated under benchtop, tissue–based, and in vivo conditions. This positions the proposed system as a functionally integrated, low–complexity alternative focused on real–time neural interfacing rather than high data rate or long–range communication.

As with other inductively coupled wireless systems operating in the HF RFID regime, the performance of the proposed implant depends on the relative orientation between the reader and implant antennas. Optimal power transfer occurs when the inductive coils are approximately parallel, whereas angular misalignment reduces magnetic coupling and available harvested power. Robustness to angular misalignment was not explicitly evaluated in this study, which focused on functional validation under nominal coupling conditions. Similar orientation–related considerations have been discussed for RF–powered implantable platforms [24]. Systems such as RFlexor exemplify this design space, and a systematic evaluation of angular tolerance is therefore left for future work.

The implant prototype validated in this study represents an early yet promising step in neuroengineering, aimed at enabling peripheral nerve recording and stimulation through a fully battery–free architecture. By eliminating the need for an internal power source, the system addresses a major limitation of conventional neuromodulatory implants related to battery replacement and device longevity. This design choice aligns with ongoing efforts to improve long–term viability and miniaturization in neurostimulation technologies, including recent developments in spinal cord stimulation and peripheral neuromodulation systems [25,26]. In this context, the in vivo experiments are intended to demonstrate functional feasibility and system–level reliability under realistic biological conditions, rather than to support population–level generalization. Further validation will require studies with larger cohorts, controlled neurophysiological injury models, and extended experimental protocols to assess system performance, stability, and functional responses under chronic conditions.

Unlike previous systems utilizing single–cuff wireless architectures, the validated prototype achieves complete battery elimination through a simplified RFID link, ensuring indefinite operational longevity without recharging [17]. Furthermore, while established frameworks for sensory feedback provide robust results, this study advances the field by demonstrating a dual–function (sensing and stimulation) platform validated through a 20 mm tissue barrier, prioritizing real–time responsiveness with minimal hardware overhead [21].

The implant placement strategy and the use of superficial extraneural cuff electrodes followed established principles for optimizing peripheral nerve signal recording [27]. The association between recorded bioelectrical signals and limb movement elicited by controlled tactile stimulation, such as the Von Frey test, further supports the adequacy of this approach for functional signal acquisition [20]. While the system does not aim to achieve fascicular–level selectivity, the results indicate that meaningful neural activity can be captured at the nerve level using a simplified and reproducible electrode interface.

The 40 µV activation threshold was defined as a conservative electronic criterion to ensure reliable triggering above the system noise floor during experimental validation. This threshold was selected to minimize false–positive detections under wireless, battery–free operation and was not intended to represent a physiological or source–specific neural threshold.

Electrical stimulation was delivered through the stimulation electrode using biphasic current pulses generated by the implant microcontroller upon receiving control commands from the external device [21]. The biphasic waveform was intentionally selected to limit electrochemical reactions and reduce the risk of tissue damage, in accordance with widely accepted practices in peripheral nerve stimulation [22]. Controlled stimulation was initially configured via Bluetooth and subsequently validated under radiofrequency operation, confirming the system’s capacity to apply reproducible and safe electrical pulses through nerve–placed cuff electrodes [21]. A key aspect of the proposed system is its ability to support adjustable stimulation parameters, enabling optimization for individual needs while adhering to established safety guidelines [15,18,19].

The observed motor responses were consistent with the functional integrity of the stimulation pathway and suggest potential applicability for future neural bridging concepts, pending further validation. RFID–based power transfer, being non–ionizing, presents minimal biological risk, and power levels were maintained within established safety standards to prevent tissue heating, supporting safe energy harvesting during operation [25]. Thermal safety is a critical constraint for implantable electronics. The observed maximum surface temperature of the implant reached approximately 28 °C during one hour of continuous benchtop operation, remaining below physiological temperature and indicating no additional thermal burden associated with RFID power harvesting. Although future iterations may incorporate more advanced signal processing or classification strategies, the current focus on peak detection ensures real–time responsiveness with minimal computational overhead, which is particularly important for battery–free implantable systems [28].

Beyond thermal behavior, additional safety considerations relevant to implantable neurostimulation systems must be acknowledged. In this study, stimulation was delivered through commercially available extraneural cuff electrodes operating within standard current and pulse parameters, and no macroscopic adverse tissue effects were observed during the experimental period. However, detailed characterization of electrode–tissue interface phenomena, including long–term impedance changes, charge density distribution, and chronic inflammatory responses, was not performed. Similarly, although the RFID–based power transfer operates at non–ionizing frequencies and demonstrated thermal stability, explicit SAR quantification was not included and remains an important aspect for future studies involving longer implantation durations and chronic use.

From a broader perspective, the proposed system should be interpreted within the context of existing wireless and battery–free neural interfaces. Prior work has demonstrated architectures capable of higher channel counts, increased bandwidth, and more advanced signal processing by incorporating active power management or local energy buffering [5,6,24]. While these approaches enable more detailed neurophysiological interrogation, they also increase system complexity and impose constraints on long–term deployment. In contrast, the present work adopts a streamlined, strictly passive RFID architecture, optimized for real–time sensing and stimulation rather than high–bandwidth data streaming or multi–site recording. These constraints reflect an explicit design choice prioritizing low–profile deployment and continuous operation, positioning the system as a robust system–level platform focused on functional neural modulation rather than high–resolution characterization.

The study validates battery–free signal capture and stimulation using commercially available cuff electrodes, which inherently limit fascicle–specific selectivity and restrict interaction to broader nerve–level regions. Despite this limitation, prior clinical and experimental studies have shown that effective functional outcomes, such as restoration of movement in fibular nerve injuries associated with drop foot, can be achieved through stimulation near the neuromuscular junction without precise fascicular targeting [29]. The present work focused on a minimal channel configuration; future applications may benefit from an increased number of acquisition and stimulation channels. Biocompatibility testing, including chronic tissue response, remains an essential step toward clinical translation. Although in vivo compatibility was not assessed in this study, the implant was encapsulated with a certified medical–grade epoxy resin approved under ISO 10993 standards [9].

It is acknowledged that advanced rechargeable implantable neurostimulators currently represent robust and clinically validated solutions for long–term neuromodulation. Nevertheless, battery–based systems inherently introduce dependency on energy storage components, which may impose constraints related to device volume, recharging logistics, and, in cases of battery failure or depletion, the potential need for surgical revision. In this context, battery–free architectures remain of interest as complementary alternatives for specific experimental, translational, and minimally invasive applications.

The integration of the implantable unit with an external robotic hand provides a system–level integration example of the proposed architecture for future neural interfacing applications. By translating acquired bioelectrical signal peaks into motor commands across a wireless RFID link and through biological tissue, the system validates the end–to–end efficiency of power delivery and data transmission in a realistic operating scenario. In terms of innovation and future use, the proposed architecture could be applied to control external devices, such as robotic hands, by associating acquired bioelectrical signals with predefined motor commands. In this context, the implant may act as a trigger to initiate and sustain specific movements, offering a potential pathway toward enhanced autonomy for amputee patients [1]. When operating as a neural bridge, the system is designed to capture neural signals proximal to a peripheral nerve lesion and automatically stimulate the nerve distal to the injury, enabling functional signal transmission across the lesion site. Although preliminary, this concept requires future fascicular–level studies and could open new possibilities for early intervention in acute peripheral nerve injuries, potentially mitigating long–term complications such as degeneration and irreversible muscle atrophy [5].

Taken together, these results support continued investigation of strictly battery–free, bidirectional neural interfaces in scenarios where long–term operation and hardware simplicity are critical. The system–level validation presented here represents a meaningful step forward toward scalable neuroengineering technologies enabled by maintenance–free, wirelessly powered implantable platforms.

## 5. Conclusions

This work provides a system–level experimental validation of a dual–function, battery–free, RFID–powered implantable platform for wireless bioelectrical signal acquisition and peripheral nerve stimulation. Across benchtop, tissue–based, and in vivo conditions, the prototype consistently achieved stable wireless powering, reliable data transfer, and reproducible functional stimulation responses under strictly passive operation, supporting TRL–6 feasibility of this architecture as a foundation for next–stage neuroengineering studies.

## Figures and Tables

**Table 1:** Benchtop Gain Validation Results. This table presents the theoretical and measured gain values for two representative amplification settings (63 dB and 93 dB), illustrating the system’s performance across the tested operational range using three device interfaces (D1–D3). It includes input and output measurements, calculated linear and decibel gains, and error margins. These data validate the core hardware performance prior to integrated system tests. (Ri: Input Resistance; Rf: Feedback Resistance; Linear Gain: Calculated ratio (no decibel scale); Total Gain (dB): Gain expressed in decibels; Error (dB): Difference between theoretical and measured gain).

	Total Gain dB		63 dB	93 dB
	Linear Gain		1412.54	44,668.36
Theory	Gain Amplifier Instrumentation		103	103
	First Stage	Ri	10,000	10,000
		Rf	3300	200,000
		Gain	1.33	21
	Second Stage	Ri	10,000	10,000
		Rf	9100	200,000
		Gain	10.2	21
	Linear Gain		1383.6	45,423
	Total Gain dB		62.82	93.15
D1	In		0.0008	0.0001
	Out		1.025	3.55
	Linear Gain		1281.25	35,500
	Total Gain dB		62.15	91
	Error (dB)		0.67	2.15
D2	In		0.0008	0.0001
	Out		0.5625	3.9125
	Linear Gain		703.13	39,125
	Total Gain dB		56.94	91.85
	Error (dB)		5.88	1.3
D3	In		0.0008	0.0001
	Out		0.015	3.56
	Linear Gain		1268.75	35,625
	Total Gain dB		62.07	91.04
	Error (dB)		0.75	2.11

**Table 2:** This table summarizes the stimulation performance results for the neural stimulation system, focusing on the tested device interfaces D1, D2, and D3. The measured parameters include output and input voltage amplitudes, converted output currents, pulse widths, frequencies, and total stimulus duration. Stimulation performance was evaluated using a 1 kΩ resistive load, and the output values were recorded using an oscilloscope and compared with the configured interface parameters. The observed results help validate the accuracy of pulse delivery, amplitude stability, and timing precision, ensuring that the stimulator operates within the specified biomedical safety and performance margins.

Device	Amplitude		Pulse Width (µs)			Frequency (Hz)			Duration
(DI)			100 µs	200 µs	250 µs	10 Hz	20 Hz	30 Hz	(seconds)
**D1**	**100 µA**	O: 812.5 mVpp = 819.05 µA I: 920 mVpp	200 µs	400 µs	500 µs	9.96 Hz–100.4 ms	19.92 Hz–50.2 ms	29.85 Hz–33.5 ms	10.040 s
	**400 µA**	O: 812.5 mVpp = 819.05 µA I: 920 mVpp							
	**660 µA**	O: 1.3188 Vpp = 1.33 mA I: 1.34 Vpp							
	**900 µA**	O: 1.781 Vpp = 1.80 mA I: 1.78 Vpp							
	**1200 µA**	O: 2.391 Vpp = 2.41 mA I: 2.27 Vpp							
**D2**	**100 µA**	O: 243.8 mVpp = 245.77 µA I: 295 mVpp	200 µs	404 µs	502 µs	9.94 Hz–100.6 ms	19.88 Hz–50.3 ms	29.85 Hz–33.5 ms	10.080 s
	**400 µA**	O: 762.5 mVpp = 768.65 µA I: 900 mVpp							
	**660 µA**	O: 1.3063 Vpp = 1.32 mA I: 1.34 Vpp							
	**900 µA**	O: 1.813 Vpp = 1.83 mA I: 1.74 Vpp							
	**1200 µA**	O: 2.391 Vpp = 2.41 mA I: 2.21 Vpp							
**D3**	**100 µA**	O: 228.1 mVpp = 229.94 µA I: 300 mVpp	201 µs	402 µs	504 µs	9.92 Hz–100.8 ms	19.92 Hz–50.2 ms	29.76 Hz–33.6 ms	10.040 s
	**400 µA**	O: 825 mVpp = 831.65 µA I: 890 mVpp							
	**660 µA**	O: 1.2938 Vpp = 1.30 mA I: 1.35 Vpp							
	**900 µA**	O: 1.828 Vpp = 1.84 mA I: 1.78 Vpp							
	**1200 µA**	O: 2.391 Vpp = 2.41 mA I: 2.24 Vpp							

**Table 3:** Architectural comparison of representative wireless implantable neural systems. This system differentiates itself by providing bidirectional functionality (sensing and stimulation) in a strictly battery–free, passive RFID architecture without the need for energy buffering components.

Reference	Powering Method	Energy Buffering	Functionality	Validation Level
Kim et al. [6]	Battery–free wireless	No	Sensing	Review/Conceptual
Wang et al. [5]	Battery–free wireless	Minimal	Sensing	Peripheral nerve (acute)
Williams et al. [21]	Wireless (active)	Yes	Sensing + Stimulation	Bench
Nelson et al. [24]	RF–powered wireless	Minimal	Sensing	Review/System–level analysis
This work	Passive RFID	No	Sensing + Stimulation	Bench + Tissue + In vivo

## Data Availability

The data presented in this study are available in this article and Supplementary Materials. Further inquiries can be directed to the corresponding author.

## Supplementary Materials

The following supporting information can be downloaded at: https://www.mdpi.com/article/10.3390/s26030954/s1; Figure S1: Simulated 1 kHz sine wave used during bench testing; Figure S2: Oscilloscope capture of a 1 kHz signal applied to the input stage during bench testing; Figure S3: Experimental setup for bench testing with generator, attenuator and devices; Figure S4: Test setup for stimulus circuit validation with 1 kΩ load; Figure S5: Measurement of biphasic stimulation pulse width; Figure S6: Measurement of total stimulation duration; Figure S7: Prototype evaluation in tissue-mimicking environment; Figure S8: System-level integration test transmitting signal to robotic hand; Figure S9: Oscilloscope display of stimulus signal and electrode current; Figure S10: Recorded electrophysiological signal during paw movement stimulation; Table S1: External device distance of 0 mm; Table S2: External device positioned at 3 mm; Table S3: External device positioned at 20 mm; Table S4: External device positioned at 22 mm; Table S5: LED communication responses for different tissue setups; Table S6: Raw evoked response amplitudes recorded in animal tests.
