# Supplementary material for: Experimental Validation of a Battery-Free RFID-Powered Implantable Neural Sensor and Stimulator
Source: Sensors (Basel). 2026 Feb 2;26(3):954. doi: 10.3390/s26030954 (PMC12899926; doi:10.3390/s26030954)
Supplement: Supplementary file 1 [file sensors-26-00954-s001.zip › sensors-4110079-supplementary.pdf]

Supplementary Material

Experimental Validation of a Battery-Free RFID-Powered Implantable Neural Sensor and Stimulator

This Supplementary Material provides detailed experimental procedures and extended validation data supporting the Methods section of the main manuscript, with emphasis on engineering reproducibility and system-level verification. It includes additional experimental data from benchtop tests, biological tissue assessments, and extended details of the in vivo validation. The tables and figures are referenced in the main manuscript to guide the reader through the validation workflow and avoid redundancy between sections.

Throughout this Supplementary Material, the implantable device is referred to as ID, and the external unit is referred to as DAPS.

Overview of Validation Data

This section details the benchtop validation of both the signal acquisition and stimulation functionalities of the implant prototype. The tests were designed to assess the device's ability to reliably capture signals and deliver controlled electrical stimulation across varied configurations. Key parameters—amplitude, pulse width, frequency, and duration—are documented.

Each table and figure specifies both the configured parameters and the corresponding measured outputs to document the system's accuracy and stability. Oscilloscope captures include annotated cursors highlighting key measurements for clarity. Performance consistency for both acquisition and stimulation was verified during bench testing.

1. Bench Tests:

Evaluation of the operation of the neural sensor and stimulator in a controlled environment, verifying the reception and transmission of electrical signals.

The boards were individually tested using a signal generator to simulate the nerve signal. The generator was set to Burst mode (500 cycles and 2-second interval) with a sinusoidal signal of 1 kHz frequency (to reproduce the nerve signals in a controlled manner). The signal amplitude was then varied to utilize the full dynamic range of the microcontroller input, taking care to avoid saturation. The generator output was connected to a 60 dB attenuator (1000 times attenuation), which, in turn, was connected to the capture electrodes. In the developed interface, different gain values were selected, and the amplitude of the signal delivered to the microcontroller was analyzed on an oscilloscope through test point TP5.

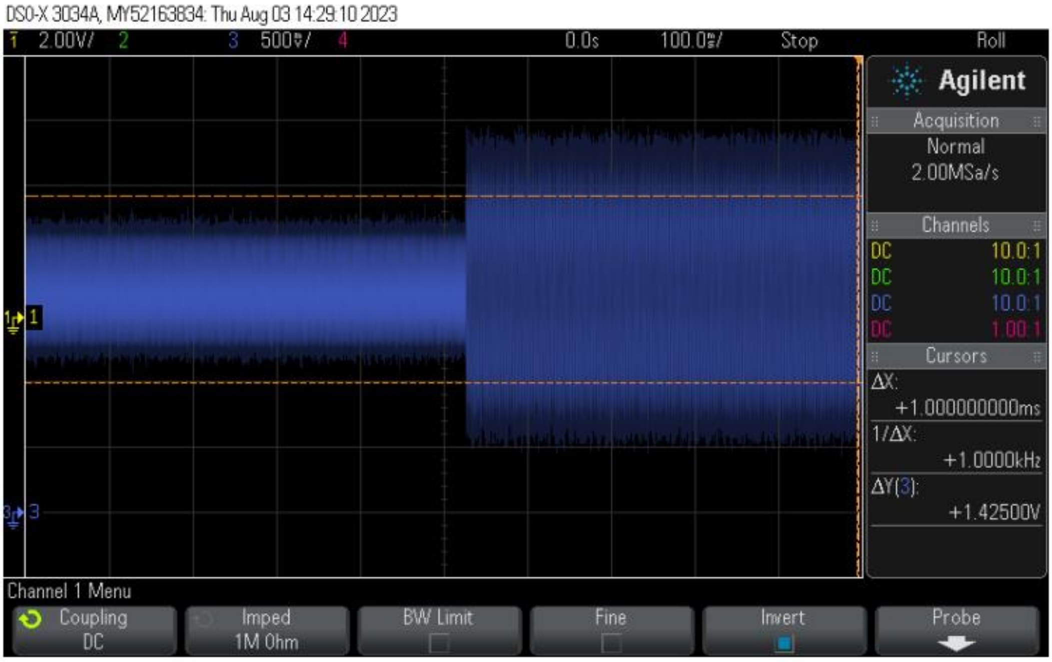

Figure S1. Simulated 1 kHz sine wave used during bench testing to verify signal integrity through the acquisition path. This waveform served as a functional input to test the basic signal capture capability of the system.

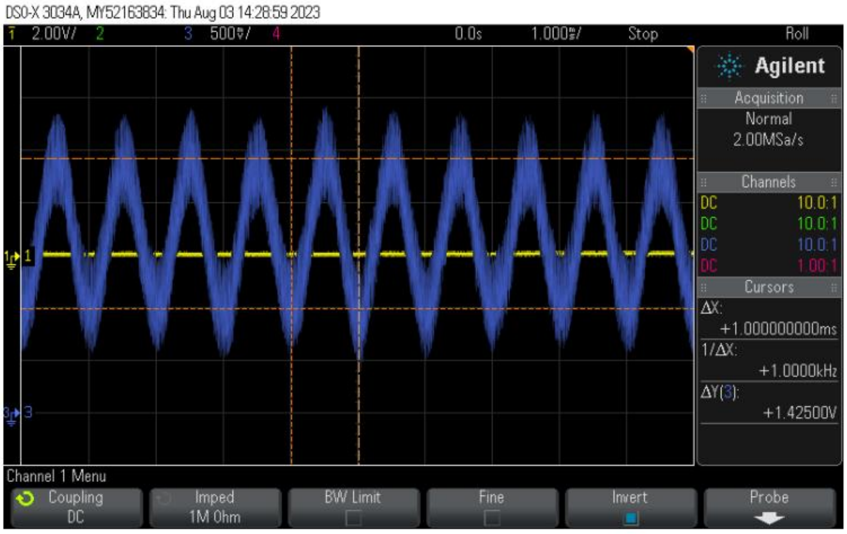

Figure S2. Oscilloscope capture of a 1 kHz signal applied to the input stage during bench testing. This example demonstrates waveform preservation through the analog acquisition chain under controlled conditions.

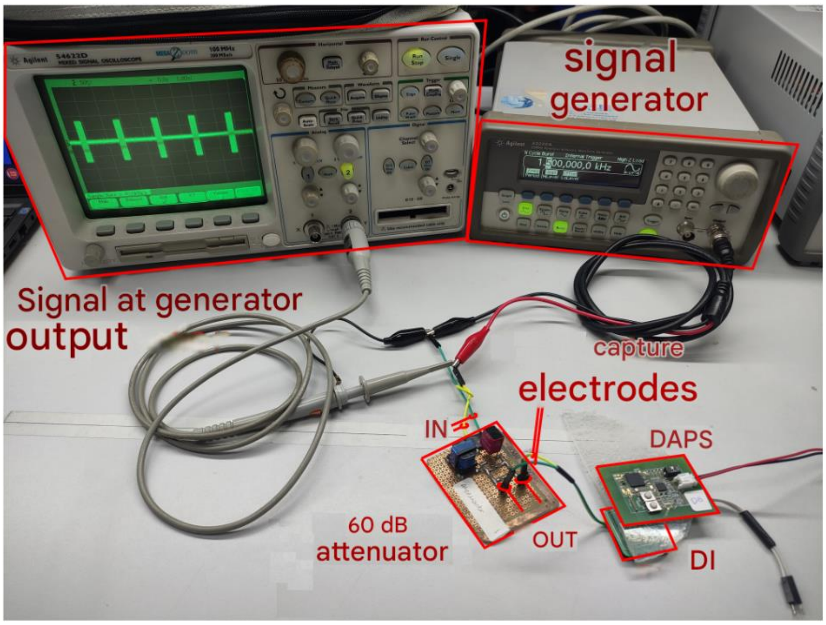

Figure S3. Experimental setup for bench testing, including the signal generator, 60 dB attenuator, capture electrodes, implantable device (DI), external device (DAPS), porcine tissue positioned between them, and an oscilloscope for signal validation.

General Testing Routine

Amplitudes were analyzed across the entire gain range available in the interface, starting with an initial stimulus amplitude of 800 mVpp. It was observed that as the gain increased, the amplitude needed to be reduced to prevent signal saturation.

The Implanted Device board was initially tested without a capacitor on the VCC of the switch, resulting in a higher error value. Adding the capacitor made the values more consistent. Additionally, it was evaluated whether the gains would maintain similar behavior when the Implanted Device board was soldered to the antenna with the proper spacing.

Stimulus Circuit

To validate the stimulus circuit, the parameters were configured in the interface, and the stimulus was applied to a 1 kΩ load. The frequency, duration, pulse width, and current intensity were measured using an oscilloscope (tests performed at Inatel’s laboratory, results obtained by measuring the voltage across the load), allowing comparison with the configured data. The measured results were also observed and compared within the interface.

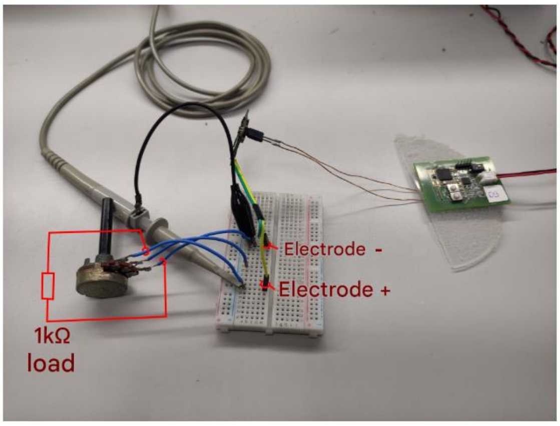

Figure S4.

Test setup for stimulus circuit validation. A 1 kΩ load is connected to the stimulus electrodes, and the voltage across the load is measured to assess circuit performance.

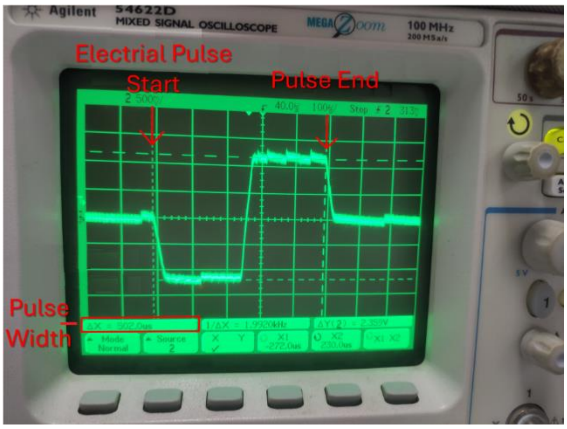

Figure S5.

Oscilloscope trace illustrating the measurement of biphasic pulse width. Test condition: Configured pulse width 502 μs. The cursors indicate the start and end of the pulse. This figure demonstrates the accuracy of the implant's stimulation pulse width under bench testing.

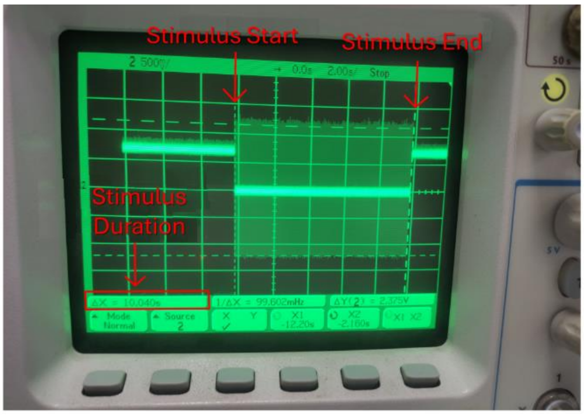

Figure S6.

Oscilloscope capture showing the measurement of total stimulus duration. Test condition: Configured duration 10 seconds; measured duration 10.04 seconds. The cursors highlight the full stimulation period. This figure demonstrates the implant's ability to maintain consistent stimulation over the programmed duration during bench testing.

General Testing Routine

The parameters for the test were varied and then compared with the interface: amplitude at 100 μA, 400 μA, 660 μA, 900 μA, and 1200 μA; duration of 10000 ms; frequency at 10 Hz, 20 Hz, and 30 Hz; pulse width at 100 μs, 200 μs, and 250 μs. A 1 kΩ load (~992 Ω measured on the multimeter) was used.

2. Proof-of-Concept Validation:

Initial Hardware Characterization

Detailed theoretical versus measured gain and stimulation results for the amplifier stages are provided in manuscript Tables 1 & 2, confirming system accuracy at the hardware level prior to integrated system and biological testing. These data validate the core hardware performance prior to advancing to integrated system and biological testing.

Bench Tests with Biological Tissues

To assess the device's response in an environment closer to real-world conditions and evaluate the influence of tissue composition on signal transmission.

A setup was assembled to validate the communication distance between the DI (Implantable Device) and DAPS (External Device). Initially, tests were conducted in air as the transmission medium, with distance variations of 0mm, 20mm, and 22mm.

Measurement points:

- Current at inductor L1 (indicating total power consumption at 3.3V – microcontroller + DC/DC converter).
- Signal generator in burst mode: 200 mVpp, 1.5 kHz.

Subsequently, additional tests were performed to evaluate the communication distance between the DAPS and DI in different media. In these tests, the DI was placed inside a 3D-printed prototype case, while the DAPS was housed in the 3D-printed PLA case. The transmission medium alternated between air and a porcine tissue sample.

Fresh biological tissues, including muscle and adipose tissue, were used to replicate different electrical conductivity conditions. The device was implanted at different depths within the tissues, and signals were recorded.

Test with air as the medium: Implantable Device board + 2A antenna connected via the External Device (DAPS) header.

	Idle	Stimulus 1200uA	Capture 63 dB	Capture 93dB
**Current**	0,51mA	2,85mA	2,42mA	3,15-3,50mA
**Voltage 3.3 V**	3,30 V	3,30 V	3,30 V	3,30 V

Table S1. External device distance: 0 mm (in direct contact).

	Idle	Stimulus 1200uA	Capture 63 dB	Capture 93dB
**Current**	0,52 mA	2,85 mA	2,45 mA	3,15-3,65 mA
**Voltage 3.3V**	3,28 V	3,27 V	3,27 V	3,26 V

Table S2. External device positioned at 3 mm from the implantable device.

	Idle	Stimulus 1200uA	Capture 63 dB	Capture 93dB	Green Positioning
**Current**	0,48 mA	2,75 mA*	2,27-2,54 mA	3,12-3,64 mA	3,40 mA
**Voltage 3.3V**	3,27 V	3,26 V	3,26 V	3,26 V	3,26 V

*Without capacitor C18 (filter on the analog input of the stimulus feedback), the consumption dropped to 2.11 mA.

Table S3. External device positioned at 20 mm from the implantable device.

*Capacitor C18 serves as an analog filter (low-pass) in the stimulus feedback loop, used exclusively for internal circuit stabilization during bench tests. It does not affect the physiological signal acquisition pathway. For neural signal acquisition, no analog filtering was applied; only digital filtering was implemented as described in the Methods section of the main manuscript. The constant current source exhibits a leak current of less than 1 μA under normal operating conditions.

	Idle	Stimulus 1200uA	Capture 63 dB	Capture 93dB *150mVpp	Green Positioning
**Current**	0,48 mA	3,32 mA*	2,60-2,70 mA	3,03-3,10 mA	3,03 mA
**Voltage 3.3V**	3,27 V	3,02 V	3,26 V	3,24 V	3,26 V

*It was necessary to reduce the generator signal voltage. With the voltage of 220mVpp and maximum gain, the voltage reduces drastically and the capture does not work.

Table S4. External device positioned at 22 mm from the implantable device.

Summary Statement - Across the tested configurations, waveform integrity was preserved, with no saturation or instability detected under the evaluated acquisition and stimulation settings. The measured outputs consistently matched the expected values within acceptable error margins, confirming the device's robust performance and configurability under varying stimulation and acquisition parameters.

Test using porcine tissue as the medium: Implantable device board + 2A antenna connected via the External Device (DAPS) header

Porcine tissue was chosen due to its structural and physiological similarities to human tissue. This table presents the results of communication tests between the DI (Implantable Device) and the DAPS (External Device) *utilizing* porcine tissue, designed to simulate *in vivo* conditions. Different tissue configurations and total distance were tested to assess the impact on communication, *as indicated* by the LED color.

LED Color Legend: Green: Communication successful; Red: Communication failed; Orange: Communication with weak signal.

Setup	Porcine tissue sample (10 mm) + DI case (2.3 mm) + DAPS case (2.7 mm)	Porcine tissue sample (15 mm) + DI case (2.3 mm) + DAPS case (2.7 mm)	Leather (2mm) + Porcine tissue sample (10mm) + DI Case (2.3mm) + DAPS Case (2.7mm)	Porcine tissue sample with leather (10mm) + DI Case (2.3mm) + DAPS Case (2.7mm)	Porcine tissue sample (15mm) + Leather (2mm) + DI Case (2.3mm) + DAPS Case (2.7mm)
**Board**	**Total Distance 15mm**	**Total Distance 20mm**	**Total Distance 17mm**	**Total Distance 15mm**	**Total Distance 22 mm**
	**LED Color**				
**P1**	Green	Green	Green	Green	Green
**P2**	Green	Green	Green	Green	Red
**P3**	Green	Green	Green	Green	Orange

Table S5. LED color responses for different material setups and total distances between the implantable device (DI) and the external device (DAPS). Each setup includes varying thicknesses of porcine tissue and leather, with the total distance specified in millimeters.

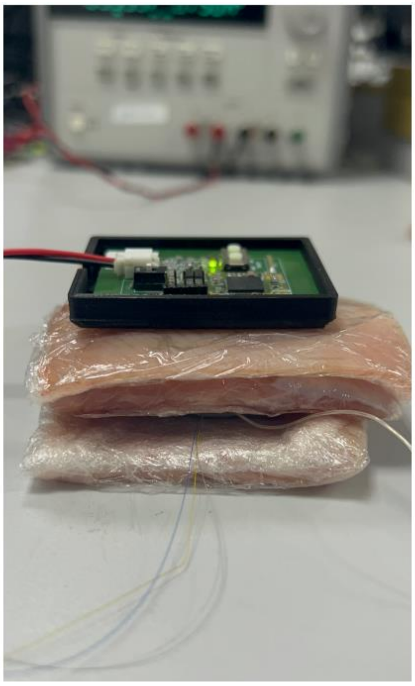

Figure S7.

Experimental setup for evaluating the prototype's performance in a tissue-mimicking environment. The image shows the device placed on layered porcine tissue, simulating realistic conditions for signal transmission and validation.

3. System-Level Integration Testing

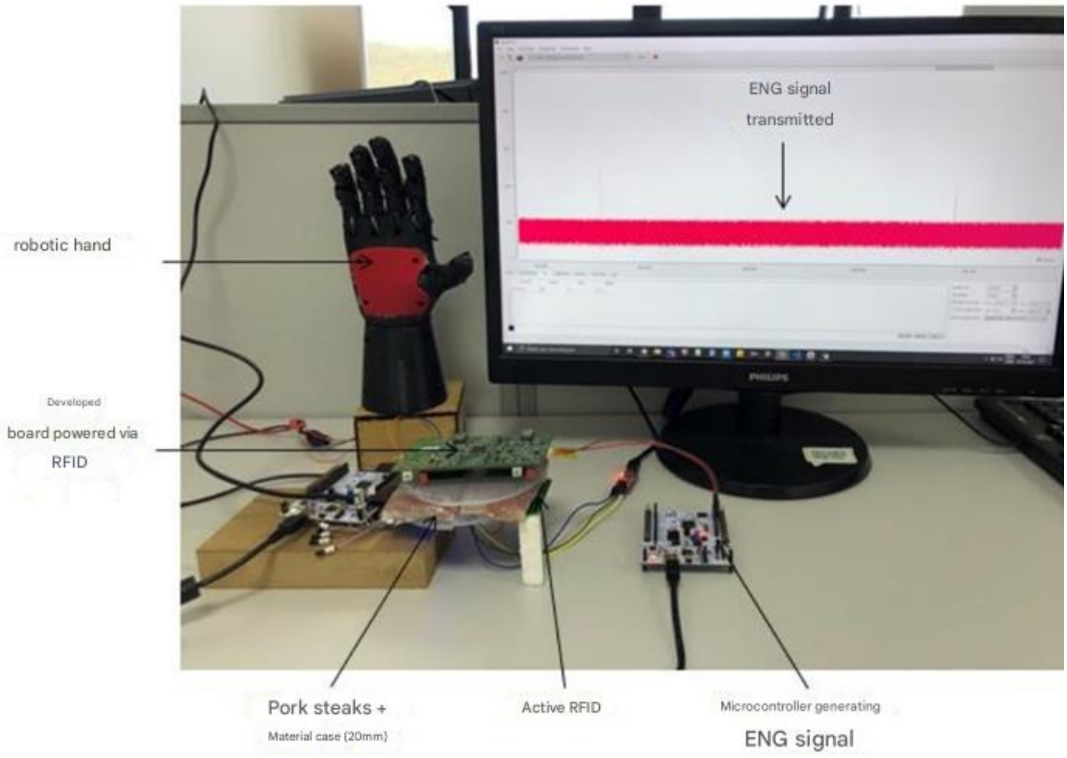

Figure S8. Proof-of-Concept (PoC) setup for integrated firmware (FW) and hardware (HW) validation. The developed board, powered via RFID, transmits an ENG signal to a robotic hand. The signal is generated by a microcontroller and propagates through a 20 mm layer of pork tissue, mimicking a biological medium for testing signal transmission and system functionality.

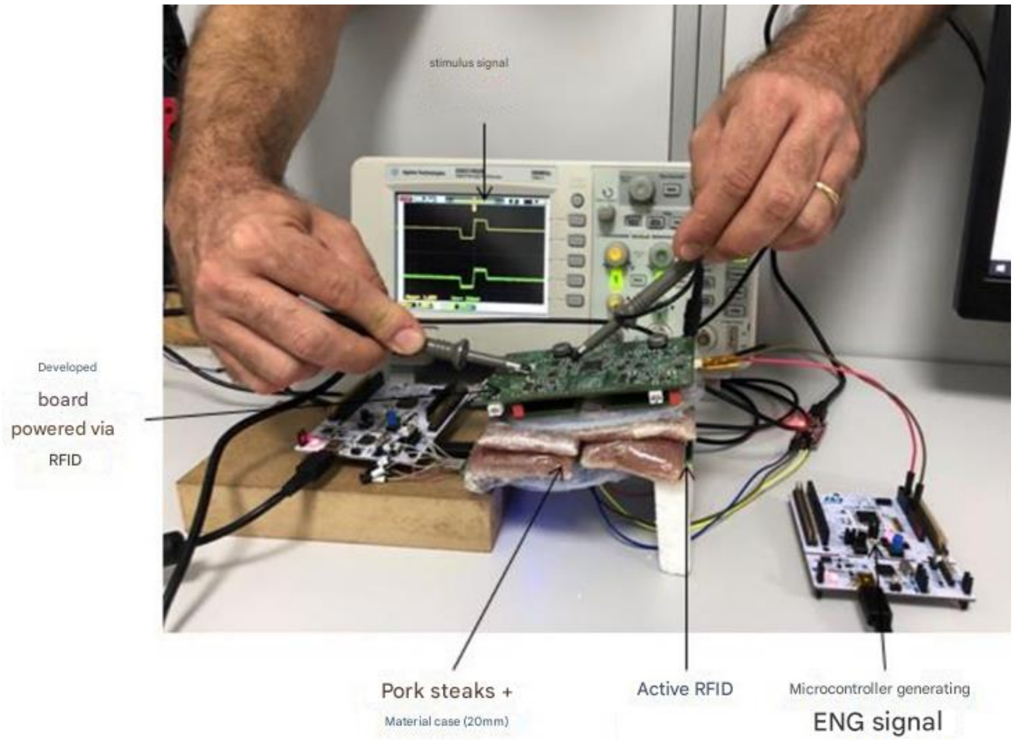

Figure S9. Oscilloscope display showing the stimulus signal and electrode current measurement during system validation. The developed board, powered via RFID, delivers a microcontroller-generated stimulus that propagates through a 20 mm layer of biological tissue. This setup is used to assess signal integrity, power efficiency, and overall system performance in a simulated implantation scenario.

4. Integrated Functional Validation: Behavioral Response and Real-Time Signal Acquisition.

To complement the technical performance data, an integrated functional validation was performed to correlate biological behavior with electronic signal detection. This test demonstrates the system's ability to operate in a functionally integrated scenario, where a mechanical stimulus (tactile) results in an observable physiological response (paw extension) and a simultaneous bioelectrical recording. As shown in Figure S10, the battery-free implant successfully captured signal peaks exceeding the pre-defined activation threshold of 40 μV, maintaining data integrity during animal movement. This qualitative assessment supports the quantitative findings of signal stability presented in the main manuscript.

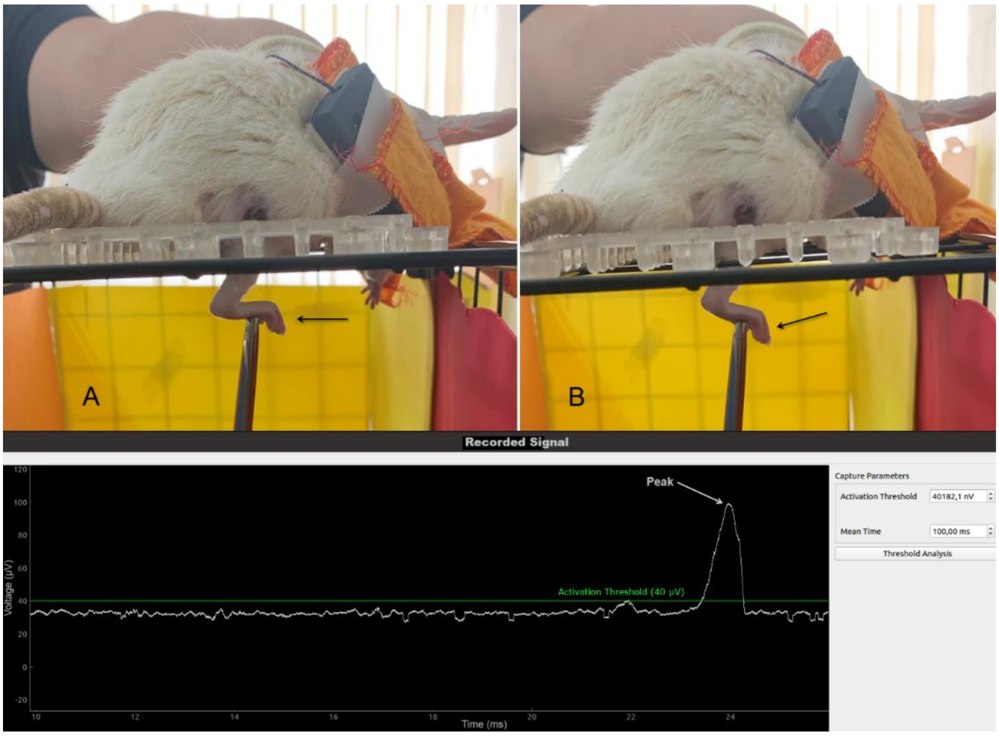

Figure S10: Recorded electrophysiological signal corresponding to paw movement during tactile stimulation. (A) Photograph showing the rat's hind limb at rest, indicated by the arrow. (B) Photograph showing paw extension following tactile stimulation, also indicated by the arrow. The lower panel displays the corresponding voltage signal recorded by the battery-free implant. The signal peak—potentially associated with the observed paw extension—is marked by the arrow and labeled 'Peak'. Horizontal lines indicate the user-defined 'Activation Threshold' (40 μV), which is used by the firmware for real-time signal detection and do not correspond to physiological threshold. The x-axis represents time (ms), and the y-axis represents voltage amplitude (μV).

During the in vivo evaluation period, experimental sessions were conducted on scheduled days over a three-month monitoring window. Animals were evaluated individually, and each session comprised device alignment, system activation, and a single controlled stimulation or acquisition event. The external unit was temporarily positioned parallel to the implanted device during active testing to enable wireless power transfer and data communication and was removed immediately afterward. Session duration, including animal handling and device setup, ranged from approximately 30 to 60 minutes.

To assess the functionality of the recording interface under biologically relevant conditions, controlled tactile stimulation was applied to the plantar surface of the hind paw using a mechanical stimulus comparable to a Von Frey filament. This approach was used to elicit reproducible motor

responses while enabling temporal association between tactile input and recorded bioelectrical activity. The procedure was selected as a functional validation method rather than a quantitative neurophysiological assessment.

Electrical stimulation was delivered through the stimulation electrode using biphasic current-controlled pulses generated by the implant microcontroller upon command from the external unit. Biphasic waveforms were selected to minimize electrochemical reactions at the electrode–tissue interface and reduce the risk of tissue damage, consistent with established practices in peripheral nerve stimulation.

Specific absorption rate (SAR) testing was not performed, as it was outside the scope of the present study, which focused on functional and technical validation of the prototype. The RFID operating frequency employed is widely regarded as safe according to established literature. During extended laboratory operation, continuous energy harvesting for acquisition and stimulation over one hour resulted in a maximum device temperature of 28 °C, indicating thermal stability under prolonged operation during benchtop tests.

At the conclusion of the three-month evaluation period, all animals remained alive and were returned to institutional housing facilities. No terminal procedures or euthanasia were required, as the study did not involve histological or biochemical endpoints. All procedures complied with the AVMA Guidelines for the Euthanasia of Animals (2020) and Brazilian CONCEA and CEUA regulations (protocol 22538137).

5. Raw Data Table – Evoked Responses in Animal Tests

To standardize signal inclusion criteria and reduce variability associated with noise and transient artifacts, a minimum amplitude threshold was defined for bioelectrical signal analysis. Signals exceeding 40 µV were considered valid for further evaluation, as this level was consistently above baseline noise observed during benchtop and tissue-based testing under identical acquisition settings. This threshold was selected as an operational criterion to ensure that recorded events reflected reproducible bioelectrical activity temporally associated with controlled stimulation, rather than isolated fluctuations or background interference. The threshold was applied uniformly across all experimental sessions and animals and was not used as a physiological cutoff, but solely as a technical filtering parameter for signal consistency.

	Animal ID	Trial #	Evoked Response Amplitude (µV)	Threshold (µV)
1	Rat 1	1	88	40
2	Rat 1	2	98	40
3	Rat 1	3	60	40
4	Rat 1	4	58	40
5	Rat 1	5	70	40
6	Rat 1	6	75	40
7	Rat 1	7	62	40
8	Rat 1	8	79	40
9	Rat 1	9	82	40
10	Rat 1	10	65	40
11	Rat 1	11	80	40
12	Rat 1	12	75	40
13	Rat 1	13	55	40
14	Rat 1	14	80	40
15	Rat 1	15	70	40
16	Rat 1	16	58	40
17	Rat 1	17	65	40
18	Rat 2	1	75	40
19	Rat 2	2	60	40
20	Rat 2	3	90	40
21	Rat 2	4	88	40
22	Rat 2	5	100	40
23	Rat 2	6	81	40
24	Rat 2	7	79	40
25	Rat 2	8	71	40
26	Rat 2	9	85	40
27	Rat 2	10	85	40
28	Rat 2	11	60	40
29	Rat 2	12	55	40
30	Rat 2	13	70	40
31	Rat 2	14	80	40
32	Rat 2	15	65	40
33	Rat 2	16	60	40
34	Rat 2	17	85	40

35	Rat 3	1	65	40
36	Rat 3	2	79	40
37	Rat 3	3	71	40
38	Rat 3	4	63	40
39	Rat 3	5	79	40
40	Rat 3	6	92	40
41	Rat 3	7	87	40
42	Rat 3	8	93	40
43	Rat 3	9	71	40
44	Rat 3	10	81	40
45	Rat 3	11	110	40
46	Rat 3	12	105	40
47	Rat 3	13	75	40
48	Rat 3	14	81	40
49	Rat 3	15	85	40
50	Rat 3	16	87	40
51	Rat 3	17	95	40

Table S6. Raw data of evoked response peak amplitudes recorded during Von Frey tests across all animals and trials during signal acquisition tests.

ACKNOWLEDGMENTS: This device, patented under number BR 10 2022 010621 5 A2, was supported by funding from the Brazilian Company for Industrial Research and Innovation (EMBRAPII) and the Informatics Law (Law 8.248 of 1991).
